# How to Obtain a Mega-Intestine with Normal Morphology: In Silico Modelling of Postnatal Intestinal Growth in a Cd97-Transgenic Mouse

**DOI:** 10.3390/ijms22147345

**Published:** 2021-07-08

**Authors:** Felix Hofmann, Torsten Thalheim, Karen Rother, Marianne Quaas, Christiane Kerner, Jens Przybilla, Gabriela Aust, Joerg Galle

**Affiliations:** 1 Research Laboratories, Department of Surgery, Leipzig University, 04107 Leipzig, Germany; Karen.Rother@medizin.uni-leipzig.de (K.R.); Marianne.Quaas@medizin.uni-leipzig.de (M.Q.); Christiane.Kerner@medizin.uni-leipzig.de (C.K.); Gabriela.Aust@medizin.uni-leipzig.de (G.A.); 2 Interdisciplinary Institute for Bioinformatics (IZBI), Leipzig University, 04107 Leipzig, Germany; galle@izbi.uni-leipzig.de; 3 Institute for Medical Informatics, Statistics and Epidemiology (IMISE), Leipzig University, 04107 Leipzig, Germany; jens.przybilla@imise.uni-leipzig.de

**Keywords:** mega-intestine, postnatal development, crypt formation, Cd97, Klf4, Areg, individual cell-based model, contact inhibition of growth, crypt fission, stem cell fate, fate decision

## Abstract

Intestinal cylindrical growth peaks in mice a few weeks after birth, simultaneously with crypt fission activity. It nearly stops after weaning and cannot be reactivated later. Transgenic mice expressing *Cd97*/*Adgre5* in the intestinal epithelium develop a mega-intestine with normal microscopic morphology in adult mice. Here, we demonstrate premature intestinal differentiation in *Cd97*/*Adgre5* transgenic mice at both the cellular and molecular levels until postnatal day 14. Subsequently, the growth of the intestinal epithelium becomes activated and its maturation suppressed. These changes are paralleled by postnatal regulation of growth factors and by an increased expression of secretory cell markers, suggesting growth activation of non-epithelial tissue layers as the origin of enforced tissue growth. To understand postnatal intestinal growth mechanistically, we study epithelial fate decisions during this period with the use of a 3D individual cell-based computer model. In the model, the expansion of the intestinal stem cell (SC) population, a prerequisite for crypt fission, is largely independent of the tissue growth rate and is therefore not spontaneously adaptive. Accordingly, the model suggests that, besides the growth activation of non-epithelial tissue layers, the formation of a mega-intestine requires a released growth control in the epithelium, enabling accelerated SC expansion. The similar intestinal morphology in *Cd97*/*Adgre5* transgenic and wild type mice indicates a synchronization of tissue growth and SC expansion, likely by a crypt density-controlled contact inhibition of growth of intestinal SC proliferation. The formation of a mega-intestine with normal microscopic morphology turns out to originate in changes of autonomous and conditional specification of the intestinal cell fate induced by the activation of *Cd97*/*Adgre5.*

## 1. Introduction

Epithelial proliferation is tightly controlled. Loss of control leads to hyperplasia and tumour formation [1]. A major growth control mechanism of epithelia is the contact inhibition of growth [2,3]. Here, cell–cell and cell–matrix contacts control the onset of proliferation, rendering the cells capable of adapting their proliferation to changing cell density. In the intestine, such control is essential for embryonic development and leads to steady state regulation. The mechanism is still active in many colorectal cancer cell lines [4,5]. Its role in postnatal intestinal growth remains elusive.

During postnatal growth, the length and diameter of the mouse small intestine markedly increase, and the intestinal stem cell (SC) pool dramatically expands due to crypt fission, the process by which a parental crypt divides and produces two daughter crypts [6]. Accordingly, proliferation is strongly activated. In adult mice, permanent epithelial cell shedding at the intestinal villi still requires active proliferation [7], while SC expansion and crypt fission largely cease. Here, we look for the mechanisms that control epithelial cell fates in these periods, with a particular emphasis on proliferation.

Transgenic or knock-out mice with altered postnatal intestinal growth characteristics help to elucidate major pathways controlling this process [8,9,10]. However, the interaction between the different tissue layers, including the mucosa with the epithelium, the submucosa, which mainly consists of fibroblasts, the smooth muscle layers of the muscularis and the serosa, impedes the understanding of the regulation. Due to these interactions, the activation of a single pathway can strongly affect the growth of the entire intestine, while its repression may change only epithelial morphology or the other way around. A prominent example of such complex regulation is hyaluronic acid-induced growth changes in the intestine of postnatal mice [11,12,13]. Simplifying, we start our investigations assuming that postnatal intestinal growth originates in the expansion of the outer, non-epithelial layers of the tissue. This expansion leads to reduced epithelial cell density, loss of contact inhibition of growth and thus to enforced epithelial cell proliferation and crypt fission, that is, to adaptive growth of the epithelium. These assumptions are supported by experimental findings that postnatal hyper-activation of epithelial cell proliferation, while changing the morphology of the epithelium, does not increase intestinal length [Riehl et al., 2015, 2012], that is, does not enforce intestinal growth.

*Cd97*/*Adgre5* is a member of the unique adhesion family of G protein-coupled receptors (ADGRs), which show a bipartite adhesive/receptive structure. Transgenic mice expressing *Cd97* in intestinal epithelium (*Tg(villin-Cd97)2*, in short: Tg2 mice) develop a mega-intestine with hyper-proliferation of the transit-amplifying cells in the postnatal period, though no obvious microscopic intestinal differences exist between adult Tg2 and wild-type (WT) mice [8]. A morphological analysis of the growth process, including the quantification of the crypt and villi size, the crypt numbers and the distribution of proliferative cells in the tissue, has already been undertaken [8]. Here, we provide data on the molecular and cellular changes associated with the formation of the mega-intestine in these mice. We demonstrate that transgenic *Cd97* results in premature intestinal differentiation. This would likely reduce, not enhance, intestinal growth as seen in Tg2 mice after downregulation of the key transcription factor *Prdm1.* However, an increase in growth factor transcripts, among them the EGFR/ErbB1 ligands *Areg* and *Btc*, afterwards likely accelerates tissue growth and suppresses full intestinal maturation. These results suggest that the formation of a mega-intestine in Tg2 mice originates in growth factor-enforced expansion of the outer, non-epithelial layers of the tissue.

In order to study the epithelial response of accelerated tissue expansion, we introduce a 3D computational model of an adaptive epithelial growth process including crypt formation, SC expansion and crypt fission. This model describes the growing epithelium and part of the submucosa as self-organizing populations of individual cells that adhere to a polymer membrane and shape it via cell–matrix interactions. Growth of the muscularis, that is, tissue growth, is modelled assuming an externally forced expansion of the polymer membrane.

Simulations of postnatal intestinal growth by our model demonstrate that changes in the tissue growth rate do not affect SC expansion in the adaptive epithelium and suggest a reduced sensitivity to contact inhibition of growth to enable accelerated SC expansion as seen in Tg2 mice. We demonstrate that crypt density can control such regulation. Thus, the expansion of intestinal SCs during postnatal growth might be controlled by 3D tissue organization. Our study shows how computational models fed with appropriate experimental data can support the understanding of cell fate decisions during tissue growth processes.

## 2. Results

### 2.1. Properties of Postnatal Intestinal Growth

**Figure 1:**
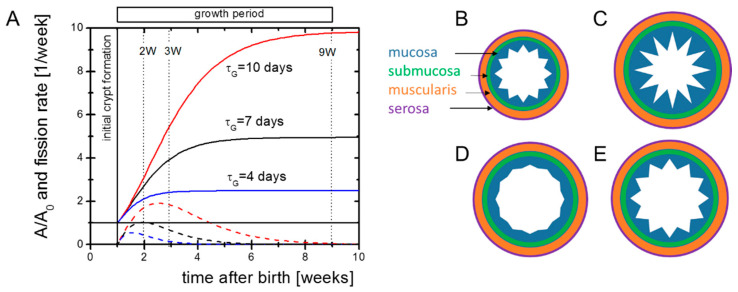
Potential growth scenarios. (**A**) Growth of the outer intestinal surface A (solid lines) according to Equation (1) and corresponding solutions for the crypt fission activity required to keep the crypt density constant (dashed lines). Our experiments aimed at characterizing the initial cellular state of the intestine and its molecular regulation in the growth phase. The dotted vertical lines indicate the time points where microarrays of intestinal samples were applied to screen gene regulation. (**B**) Initial patterning of the intestine. (**C**–**E**) Pattern of the mucosa after different growth scenarios. (**C**) Overshooting epithelial growth (e.g., after HA-administration [Riehl et al., 2012]). (**D**) Declined epithelial growth (e.g., knock-out of EGFR-ligands [Troyer et al., 2001]). (**E**) Balanced growth of the muscularis and the mucosa (e.g., in *Cd97* transgenic mice, [Aust et al., 2013]). The submucosa and the serosa are assumed to be adaptive in either case.

We start with a simple analytical view on postnatal intestinal growth to clarify our basic assumptions. In newborn mice, substantial intestinal growth starts around postnatal day seven (P7) after pre-crypt structures are formed [14]. It is active for a short period only. We approximate the change in the size of the intestinal surface by dA/dt = r_G_ exp(−t/τ_G_) A. Here, A is the surface, r_G_ the initial surface growth rate and τ_G_ the decay constant of growth. It follows:

A = A_0_*exp(r_G_*τ_G_*(1 − exp(−t/τ_G_)) (1)

where A_0_ is the initial size of the surface. Intestinal growth dynamics suggest τ_G_ in the range of one to two weeks [6]. The factor exp(r_G_*τ_G_) describes the total expansion of A. Both the external and internal areas of the mouse small intestine increase by a factor of about five [15]. Thus, one can estimate r_G_ to be in the range r_G_ = 1–2 per week. Solutions to the equation for different parameter settings are shown in Figure 1A.

During growth, the number of crypts N_C_ increases. The crypt density n_C_ = N_C_/A of neonatal and adult Tg2 mice is similar to that of WT mice [8]. Thus, we assume that intestinal surface growth and crypt fission are coupled. For constant n_c_, maximum crypt fission, activity is seen at t_mf_ = τ_G_ ln(r_g_τ_G_). Increasing r_G_ and/or τ_G_ increases the final intestinal size and shifts the time of maximum fission activity to higher values. Both changes are evident in Tg2 mice.

In the above approach, r_G_ and τ_G_ describe properties of the entire intestine. Patterning of the intestinal tissue, however, depends on properties of all tissue layers (Figure 1B–E). As the epithelial morphology is conserved in Tg2 mice, we expected an enforced growth of the outer layers in these mice and assumed that the epithelium simply adopts to these layers. To support this idea, we characterized the growing tissue experimentally (Figure 1A).

### 2.2. Accelerated Crypt Formation and Paneth Cell (PC) Specification in Tg2 Mice

**Figure 2:**
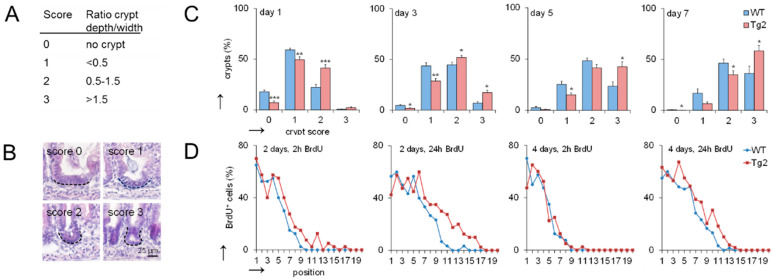
Crypt formation is accelerated in Tg2 mice. (**A**) Definition of the crypt formation score. (**B**) Representative images of the different scores in HE-stained jejunal sections. (**C**) Percentage of crypts with the different scores at postnatal days 1, 3, 5 and 7 (*n* = 5 mice/genotype, 300 crypts/mouse, mean ± SEM, * *p* < 0.05, ** *p* < 0.01, *** *p* < 0.001. (**D**) Percentage of BrdU-positive cells at a defined position along the crypt-villus axis (crypt base = 1) at P2 and P4 determined 2 and 24 h after BrdU incorporation (*n* = 5/mice/genotype/time point, *n* = 10 crypt-villus axis/mouse; mean ± SEM; WT compared with Tg2). High BrdU staining at the crypt bottom indicates that mature PCs are missing.

The mouse small intestine has narrow finger-like villi at birth. Epithelial proliferation is restricted to the intervillus domain at the base of the villi where crypt development starts [14]. We quantified the progression of crypt formation in the first postnatal week by applying a morphological score defined by the ratio between crypt length and width (Figure 2A,B). At postnatal days P1, P3, P5 and P7, crypts show a higher crypt formation score in Tg2 compared with WT mice (Figure 2C). These morphological changes are not driven by proliferation [14]. Consistently, bromodeoxyuridine (BrdU) incorporation is similar or slightly enhanced in our Tg2 mice compared with WT mice at P2 and P4 (Figure 2D).

**Figure 3:**
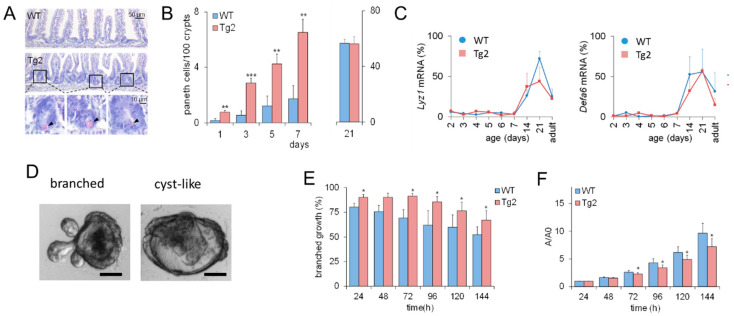
Accelerated PC development in Tg2 mice. (**A**) HE-stained jejunal sections of 7-day-old Tg2 and WT mice. PCs were identified by their typical eosinophil staining (arrows). (**B**) The number of PCs was determined in 100 duodenal and 200 jejunal crypts (HE-stained intestinal sections, *n* = 5 mice/genotype/time point, *n* = 300 crypts/mouse, mean ± SEM; ** *p* < 0.01, *** *p* < 0.001 WT compared with Tg2). (**C**) Age-dependent *Lyz1* and *Defa6* expression in jejunal samples of Tg2 and WT mice determined by qRT-PCR (*n* = 3 mice/genotype/age, normalized to *Rps29*, mean ± SD). (**D**) Growth pattern of branched and cyst-like organoids after one week of culture (scale bar: 50 µm). (**E**) Different growth properties of Tg2 and WT intestinal organoids: Percentage of SC-derived organoids of 2-week-old mice with a branched growth pattern (*n* = 5 mice/genotype, *n* = 15 organoids/mouse, mean ± SEM; * *p* < 0.05 WT compared with Tg2). (**F**) Organoids of two-week-old WT mice grew faster compared with those of age-matched Tg2 mice. The area A of the stacked cross-section of each organoid was quantified relative to that of the first time point A_0_ (*n* = 5 mice/genotype, *n* = 15 organoids/mouse, mean ± SEM; * *p* < 0.05 WT compared with Tg2).

These results imply that CD97 expression is associated with early changes in the biomechanical properties of the intestine. Based on previous computational model results [16], we expected that this would also affect intestinal cell lineage specification. Thus, we verified whether the accelerated crypt formation is accompanied by an accelerated cell specification in Tg2 mice. We focused on PCs (Figure 3A), which contribute to the formation of the SC niche [17]. In the first postnatal week, the PC number increased in both mice (Figure 3B), but the increase was about three-fold higher in Tg2 compared with WT mice. We tested whether this earlier PC specification coincides with earlier PC maturation. Until P7, markers of mature PCs (*Lyz1*, *Defa6*) were poorly detectable by qRT-PCR. Thus, either PC maturation is not accelerated in Tg2 mice, or the PC number is still too small for detection. At P14, P21, where crypts grow in length (Supplement 1, Figure S1), and in adult mice, expression of these genes is similar in Tg2 and WT mice (Figure 3C).

### 2.3. Mature Growth Characteristics of Organoids Derived from 2-Week-Old Tg2 Mice

Premature crypt formation and PC specification might be an intrinsic epithelial effect or related to changes in the submucosa of Tg2 mice. To answer this question, we applied an intestinal organoid culture. Intestinal organoids differ in their growth pattern depending on mouse donor age. Foetal organoids normally show cyst-like growth (Figure 3D and Figure S1), whereas organoids from adult mice are more often branched [18]. In organoids derived from two-week-old Tg2 mice, this transition is accelerated; that is, they grow branched more often than organoids derived from age-matched WT mice (Figure 3E). Accordingly, their growth rate is reduced compared to age-matched WT mice (Figure 3F). It is more similar to that of organoids derived from adult WT mice (Supplement 1, Figure S1, [19]). Thus, premature development is an intrinsic property of Tg2 intestinal tissue. This observation seems to be in contradiction to the development of a mega-intestine in these mice.

### 2.4. Changed Expression of Growth Factors in Tg2 Mice after Prdm1 Downregulation

**Figure 4:**
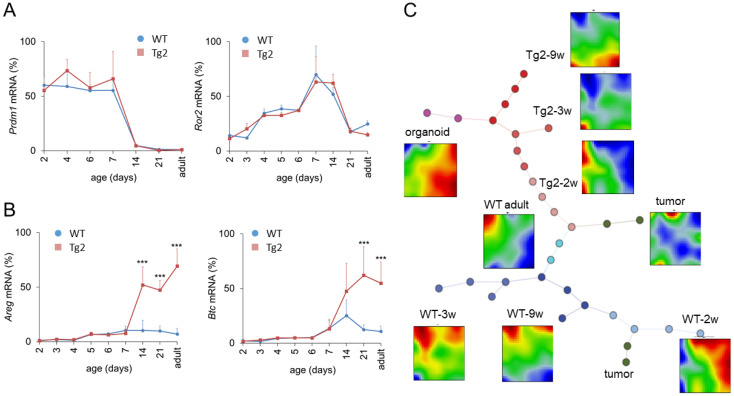
Transcriptional changes after *Prdm1* downregulation in Tg2 mice. Age-dependent gene expression in jejunal samples of Tg2 and WT mice determined by qRT-PCR (*n* = 3 mice/genotype/age, normalized to *Rps29*, mean ± SD, *** *p* < 0.001, Tg2 compared with WT). (**A**) *Prdm1* was downregulated at P14; subsequently *Ror2* became repressed in both mouse types. (**B**) At P14, the expression of the growth factors *Areg* and *Btc1* is enhanced in Tg2 mice. (**C**) SOM analysis of transcriptomic profiles of Tg2 and WT intestinal tissue. Correlation spanning tree of the transcriptomes of each sample and average SOM portraits for each sample group are shown, including samples of 2w, 3w and 9w Tg2 and WT mice, adult WT mice, organoids and intestinal tumours. In the SOM portraits, overexpressed spots appear in red, underexpressed in blue. In the spanning tree, each dot represents one analysed sample. Different dot colours indicate different sample groups. Tg2-2w samples are similar with adult WT samples supporting their premature state. In contrast, Tg2-9w samples share similarities with WT organoids, suggesting a growth factor-controlled state.

Prdm1 is a key regulator of enterocyte maturation during postnatal development. Early enlarged crypts as seen in Tg2 mice are also present in *Prdm1* knock-out mice at P7 [20]. To exclude disturbed *Prdm1* regulation in Tg2 mice, we quantified its expression. Within the first weeks after birth, *Prdm1* expression was similar in Tg2 and WT mice (Figure 4A). In accordance with [20,21], *Prdm1* was downregulated in both mice around P14. Thus, premature intestinal development in Tg2 mice is not related to an early *Prdm1* downregulation: Although crypts are formed earlier, essential gene regulatory processes associated with intestinal maturation are not accelerated. In the following, we focused on the time after *Prdm1* downregulation.

A potential scenario of Tg2-specific intestinal growth is prolonged midgut elongation. Midgut elongation in the embryo is controlled by the Wnt5A ligand Ror2 [22]. However, *Ror2* levels are similar between Tg2 and WT mice after birth and drop after *Prdm1* downregulation (Figure 4A). This suggests that embryonic growth is not prolonged in Tg2 mice and stops after *Prdm1* downregulation. In order to explain the mega-intestine formation, we next analysed growth factor regulation.

Growth factors, such as Areg and Btc, which bind to the ErbB family of receptor tyrosine kinase 1 (ERB, also named EGFR), increase the proliferative activity and regenerative potential of the intestinal epithelium [23,24] but can also stimulate the proliferation of smooth muscle cells [25]. *Areg* is a direct target of Yap1 and its induction contributes to Yap1-mediated cell proliferation [26]. In the intestine, the activation of Yap1 mediates the proliferation of smooth muscle cells and induces intestinal length growth [27,28]. Thus, we considered Areg and Btc as potential regulators of intestinal growth. We quantified their expression during the postnatal period until adulthood (Figure 4B). Until P7, *Areg* and *Btc* are weakly expressed. Afterwards, both are slightly upregulated in WT mice but strongly upregulated in Tg2 mice. This suggests that these growth factors might stimulate the enforced growth of the Tg2 intestine starting after *Prdm1* downregulation.

### 2.5. Global Gene Expression Changes in Tg2 Mice

To screen for molecular differences between Tg2 and WT mice after *Prdm1* downregulation more systematically, we analysed intestinal transcriptomic profiles starting from P14 in detail. Based on microarray data of intestinal samples of two-, three- and nine-week-old (2w, 3w, 9w) Tg2 and WT mice, we calculated gene self-organizing maps (SOM). To improve data confidence, we included data from intestinal samples of adult WT mice, intestinal tumours and WT organoids [8].

The SOM portraits differ starkly between WT and Tg2 at all developmental stages and remain different with increasing mouse age (2w-3w-9w) (Figure 4C), despite the similar morphology [8]. These differences are obvious when comparing the position and extension of over- and under-expression spots in the average group portraits.

SOM portraits of Tg2-2w mice are more like WT adult portraits than the portraits of WT-2w mice, supporting the above results on Tg2 premature differentiation. However, with increasing mouse age, Tg2 samples develop more and more similarity with WT organoids, suggesting a growth factor-controlled state similar to that of organoids [29]. This concerns enterocytes (ECs) and goblet cells (GCs) in particular. We analysed these findings in more detail (see also Supplement 1, Figures S2 and S3).

### 2.6. Specific Gene Expression in the Intestinal Epithelium in Tg2 Mice

**Figure 5:**
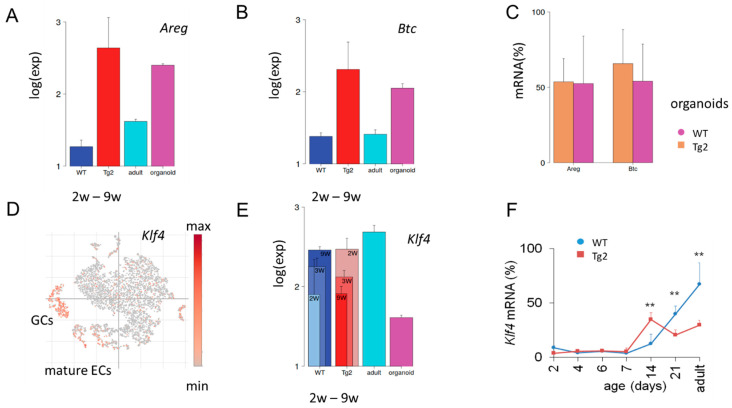
Tg2-specific expression. (**A**,**B**) Microarray data of the averaged expression of *Areg* (**A**) and *Btc* (**B**) in postnatal jejunal tissue of Tg2 and WT mice. Tg2 tissue of all young mice (2w-9w) shows similarly enhanced expression compared with WT tissue. A similar difference is seen between adult WT tissue and WT organoid samples. (**C**) *Areg and Btc* qRT-PCR levels in Tg2 and WT organoids (*n* = 5 organoid cultures/mice/genotype, normalized to *Rps29*, mean ± SD, ** *p* < 0.01). (**D**) Re-analysis of published single cell (sc)RNA-sequencing data [30]. In the adult WT epithelium, *Klf4* expression is activated in mature epithelial cells (ECs) and goblet cells (GC). Each point represents one analysed intestinal cell, which clusters according to its transcriptomic profile. (**E**,**F**) Different expression dynamics of *Klf4* in Tg2 and WT mice. (**E**) *Klf4* microarray data of intestinal jejunal samples of young (2w, 3w and 9w), adult tissue and organoids of WT mice. (**F**) *Klf4* qRT-PCR levels in Tg2 and WT mice (*n* = 3 mice/genotype/age, normalized to *Rps29*, mean ± SD, ** *p* < 0.01).

Accelerated growth of non-epithelial tissue layers in Tg2 compared with WT mice would require mechanisms of transactivation. Our qRT-PCR results on *Areg* and *Btc* suggested an enhanced secretory activity of the Tg2 epithelium. To support this hypothesis, we searched for further expression characteristics stably induced by transgenic *Cd97*. We found an entire gene cluster similarly activated in Tg2 (2w, 3w, 9w) mice and repressed in young WT (2w, 3w, 9w) mice (Supplement 1). Functional annotation shows that the cluster genes associate with the GO sets’ “response to retinoic acid” and the “immune system process”. Consistent with our qRT-PCR, we found *Areg* and *Btc* among these genes (Figure 5A,B). Their expression in Tg2 mice is activated similarly as in WT organoids, confirming an expression similarity between Tg2 tissue and organoid samples. There seems to be no additivity of this activation, as organoids from Tg2 and WT mice show similar expression (Figure 5C). While *Areg* and *Btc* are only weakly expressed in WT tissue, other genes of the cluster, such as *Cldn4* and *Tm4sf4*, are typically activated in adult secretory cells including GCs, tuft and enteroendocrine (EECs) cells (Supplement 1, Figure S4). Their increased expression further supports the idea of an enhanced secretory activity of the Tg2 epithelium.

### 2.7. Early Transient Activation of the Differentiation Marker Klf4 in Tg2 Mice

*Klf4* is expressed in post-mitotic, differentiated ECs and GCs [31] (Figure 5D) and regulates homeostasis of the epithelium [32]. In mice with conditional loss of Klf4, both the rates of proliferation and the migration of crypt cells are increased and the expression of genes encoding regulators of differentiation is reduced [33]. At P14, we found *Klf4* upregulated in Tg2 compared with WT mice, while later in development after P21, this relationship reverses (Figure 5E). We confirmed the result by qRT-PCR (Figure 5F). These findings support a premature differentiation at P14 in Tg2 mice and a suppressed maturation in adult Tg2 mice and propose Klf4 expression as a marker to monitor this fate change.

*Klf4* can be increased, and the protein can be stabilized by phosphorylated Stat3 [34]. As CD97 can activate Stat3 [35], our data provide a potential link between transgenic *Cd97* and early increased *Klf4* in Tg2 mice. How *Klf4* becomes repressed in Tg2 mice with age remains an open question.

In summary, the gene expression in Tg2 mice is consistent with the hypothesis of an enforced expansion of the outer intestinal layers in these mice and suggests growth factors as inducers.

### 2.8. A Computational Model of Neonatal Crypt Formation

From our experimental data, it remained open whether the observed increase of growth factors in Tg2 mice activates epithelial proliferation as well. We expected adaptive growth of the epithelium because intrinsic activated proliferation during postnatal growth leads to morphological changes [13] not seen in Tg2 mice. Thereby, the question arose of whether spontaneous adaptive growth of the epithelium in parallel allows for crypt fission.

**Figure 6:**
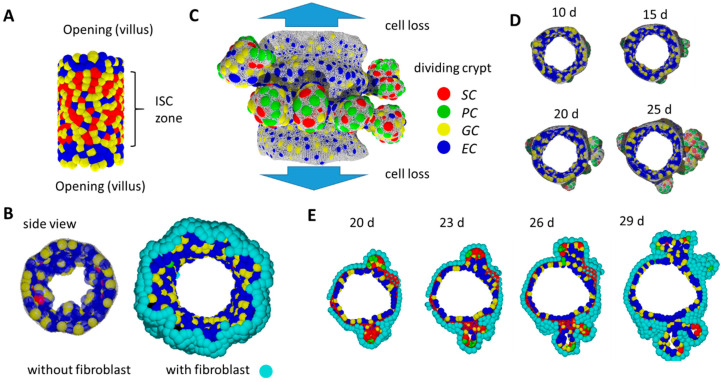
In-silico simulation of 3D crypt formation. (**A**) Initial state: *SCs* are stabilized by external Wnt. (**B**) Without a fibroblast layer beneath the growing epithelium, the tissue buckles and forms small villus-like luminal structures instead of crypts. Side views comparing systems without and with fibroblasts at the same time point. (**C**) Growing system: *SCs* are stabilized by *PCs* providing Wnt and Notch ligands. Crypts form while tissue expands. Cells moving out of the cylinder are lost. They are thought to move on the villi. Fibroblasts are not shown. (**D**) Side view of the growing tissue at different time points. Crypts form within about 2 weeks. (**E**) After that time, crypts reach a state where they undergo fission. Shown is a time series of a cross-section through the cylinder. The BM is shown in (**C**) and (**D**) only (grey net).

To answer this question, we introduced a computational model allowing the simulation of self-organized epithelial fate determination. This model comprises: (i) a population of epithelial cells adhering to a basal membrane (BM) and (ii) a population of fibroblasts adhering to the opposite side of the BM. The modelled epithelial population contains stem cells (*SCs*; *in the following*, *italics indicate model cells*) that can proliferate permanently. *SCs* maintenance requires high Wnt and Notch activity. If these activities decrease, *SCs* change their fate and specify into *PCs*, *GCs* and *ECs* (Figure 6C) [36]. Thereby, *PC* specification requires a positive threshold curvature of the tissue. Epithelial cells have proliferative capacity until they become terminally differentiated. Fibroblasts do not change their fate.

The BM is represented by a network of semi-flexible polymers. Allowing the network to re-organize, the in silico intestinal mucosa can change shape according to cell–cell and cell–network interactions. Details of the model are explained in Supplement 2.

We started our simulations with a cylindrical tissue of fixed length representing inter-villus junctions. External forces induce permanent radial growth of the cylinder with rate r (Supplement 2, Figure S5). Epithelial cells completely cover the inner, fibroblasts the outer, surface of the cylinder. Cell proliferation induces pressure in the system and cells move towards sinks in the pressure. Such sinks are localized at the openings of the intestinal cylinder. Cells crossing the openings are removed and therefore the pressure drops to zero. We assume that these cells actively migrate onto the villi [37] or follow a contraction of the differentiating villus epithelium [38]. The ‘cell turnover’ onto the villi per time unit corresponds to the pressure distribution that establishes in the system. If the turnover is blocked, cell density and thus pressure increases, and cell production becomes limited by the radial growth. For r = 0, constant pressure establishes where all cells undergo contact inhibition of growth.

During an initial simulation period, *SC* maintenance is supported by external Wnt in the central part of the tissue (Figure 6A), consistent with high Wnt activity in the inter-villus regions of mice after birth [39]. Specification of *PCs*, which provide Wnt, cannot occur in the initial tissue configuration because local curvature is too low. Only, over time, the re-organization of the polymer network enables high local curvature and thus *PC* specification. External Wnt is switched off if a threshold number of *PCs* (N_PC_ = 20) has been specified. Subsequently, *SCs* gain Wnt from these and further established *PCs* only. We consider this time point as the starting point of our postnatal growth simulation (t = 0).

### 2.9. Basic Model Simulations of Crypt Formation

For our simulation studies, we applied parameter sets from our organoid model [16]. For simplicity, fibroblasts are modelled like *ECs* (Supplement 2). Notably, the self-organizing epithelial cell population alone tends to form small villus-like structures instead of crypts (Figure 6B). The fibroblast layer on the opposite site of the BM compensates this trend, and tissue shape changes preferentially occur due to specific biomechanical properties of the BM in contact with *PCs*.

**Figure 7:**
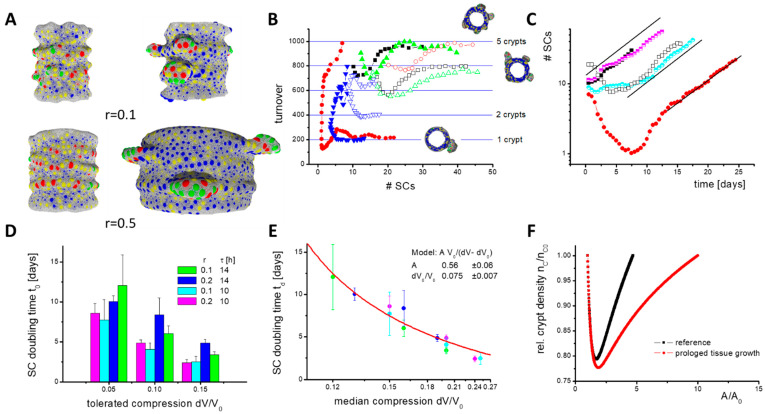
Constant cell turnover during *SC* expansion. (**A**) Growth phases: Shown are snapshots of the initial (left) and expansion phase (right) from simulations with different tissue growth rate r. Fibroblasts are not shown. Crypts expand asymmetrically. Colour code: SCs (red), PCs (green), GCs (yellow), ECs (blue). (**B**) Cell turnover onto the villi. In the initial phase, the number of *SCs* can decrease. Afterwards, SCs start expanding and a stable turnover establishes, which depends on the number of crypts formed. Shown are results of individual simulations (r = 0.1). For minimal cell cycle time τ = 14 h (open symbols), more *SCs* are required to stabilize the turnover than for τ = 10 h (filled symbols). (**C**) SC expansion. In the expansion phase, *SC* numbers grow exponentially. Similar expansion is seen with r = 0.1 (filled symbols, simulations as in (**B**)), r = 0.2 (half-filled symbols) and with blocked turnover (open symbols). (**D**) While changes of τ and of the tolerated compression dV/V_0_ change the SC doubling time t_d_, changes of r do not. The rates have been averaged over 5 simulations (error: sd). (**E**) t_d_ inversely depends on median compression of the cells dV_0_/V_0_ (colours as in (**D**)). In our model, *SC* expansion vanishes for dV_0_/V_0_ ≤ 0.075. Line: data fit. (**F**) Results of the analytical model of tissue growth with a density-dependent fission rate r_M_(1 − n_C_/n_CE_). Enforced adaptive growth reduces the crypt density. This implies higher *SC* turnover. Calculations were started with n_C_ = n_CE_. The shortest *SC* doubling time t_d_ realized is about 1 week (r_M_ = 5/week).

Our simulations demonstrate that crypts form, expand and undergo fission in parallel to tissue expansion, with and without blocked turnover onto the villi and at a wide range of growth rates r (Figure 6C–E, Supplement 2, Figure S6). For the chosen parameters, the formation of crypts takes about two weeks. Afterwards, crypts reach a size where they start fission (Video A). Fission is induced by separating *PC* clusters and includes fibroblast movement similar to that of mice [40,41]. Most of the crypt fission events take place within cross-sections of the cylinder as crypts expand asymmetrically due to radial growth (Figure 7A). In our simulations, the fission process takes more than an additional week of development. A statistical analysis of this process is therefore out of reach even with high computational power (see Materials and Methods).

Before crypt fission, two growth phases occur (Figure 7A). In the initial phase after the Wnt switch, the number of *SCs* decreases because only those *SCs* remain that are stabilized by *PCs* (Figure 7B). *SCs* tend to switch into epithelial lineages (*EC*, *GC*) after external Wnt is exhausted. Afterwards, in the expansion phase, the *SC* numbers increase, because the BM in contact with *PCs* expands under the pressure induced by proliferating cells. This leads to specification of new *PCs*, as they are required for *SC* maintenance. Consequently, a larger part of the BM expands and the number of *SCs* increases further. This positive feedback explains why niche cell numbers (*SCs* and *PCs*) grow exponentially with a constant doubling time t_D_ (Figure 7C).

During *SC* expansion, a stable cell turnover onto the villi establishes although the *SC* number steadily increases, that is, a defined number of cells N_T_ is released per crypt that is shared between the growing inter-crypt surface and the villi. This is a result of the *SC* niche pressure, which is induced by the force required to push cells out of the crypt. Reaching a threshold pressure, cells start undergoing contact inhibition of growth, and turnover cannot further increase. To reach a stable cell turnover, N_T_, a minimum number of *SCs* per crypt is required, which depends on their cell cycle time. For a longer cell cycle time, more *SCs* are required to reach a stable cell turnover (Figure 7B). They ensure similar cell production and, accordingly, longer cell cycle times do not result in a lower turnover.

Lower turnover is observed when the radial growth rate r is increased. In this case, more and more cells are required to cover the inner surface of the cylinder. This reduces the pressure in this part of the tissue and thus the number of cells moving onto the villi. Therefore, without additional changes, villi would shrink with increasing radial growth rate r. Contrarily, increasing r does not affect the niche pressure or the *SC* doubling time t_D_ (Figure 7D). This might change if the radial tissue growth requires cell production above N_T_(r = 0). The simulation of such a fast expanding system unfortunately exceeds our current computational power.

In summary, the model suggests that the expansion of the intestinal SC population, a prerequisite for crypt fission, is largely independent of the tissue growth rate.

### 2.10. Simulation of Accelerated Crypt Formation

For a limited time, the increased cell production required during tissue growth can be provided by the crypts if their production N_T_(r = 0) is initially not exhausted, as in the case of blocked turnover onto the villi. However, over the entire growth process, stable cell production per unit tissue area can be ensured only by keeping a defined crypt density. Thus, new crypts have to form via fission. To ensure constant crypt density for increasing r, the crypt size for fission (Figure 6E) has to be reached within a shorter time; *SC* niche expansion must become faster (fission rate = 1/*SC* doubling time). A straightforward way to accelerate niche expansion is to increase the niche pressure.

Blocking turnover increases the density and pressure in the cylinder region but has only a marginal effect on the niche pressure. Accordingly, the *SC* doubling time t_D_ remains constant when blocking the turnover (Figure 7C). Shortening the *SC* cycle time τ decreases their doubling time t_d_ (Figure 7D), that is, higher pressure establishes regardless of the same sensitivity to contact inhibition of growth. Here, the increased pressure results from cells that leave the G0 phase and start growing. The faster they grow, the faster neighbouring cells are pushed away. At the crypt bottom, this applies also to neighbours that stay in G0. A dynamic pressure to move them, which increases with decreasing cycle time τ, adds to the static pressure to keep them quiescent. However, epithelial cells are much more flexible in their shape than in their in silico models, which dampens this effect. Thus, we consider such effects in mice as minor.

An immediate increase of the niche pressure can be achieved by decreasing the *SC* sensitivity to contact inhibition of growth such that they can proliferate at a higher niche pressure. This pressure then establishes and niche expansion accelerates. We modelled such a scenario by increasing the relative compression that is tolerated by the cells, dV/V_0_. When plotting the *SC* doubling time t_d_ over the median compression of the *SCs*, which is proportional to the pressure in the niche, an inverse dependency between both is observed. A fit of the data allows estimating the median compression where crypt growth vanishes, that is, t_d_ goes to infinity (Figure 7E). Importantly, an intestine with WT morphology can only develop if the rate of tissue growth and *SC* expansion (crypt fission) are synchronized. A specific synchronization in Tg2 mice is unlikely, suggesting a crypt density-controlled mechanism that acts similarly in both WT and Tg2 mice.

Following this idea, we took up our simple analytical model. We assumed crypt density-dependent fission activity: dN/dt = c(n_c_)N, with a rate c(n_c_) = r_M_ (1 − n_C_/n_CE_), where n_CE_ is the equilibrium crypt density. As c(n_c_) = 1/t_d_, this is synonymous with a crypt density-controlled *SC* expansion. Replacing in Equation (1) ‘r_G_ A’ with ‘r_C_ N_C_’ allows us to study the growth process under this control mechanism. Here, r_M_ and r_C_ are constants describing the maximum fission rate and the surface production rate per crypt, respectively. Initially, the crypt density drops, but afterwards it reconstitutes in parallel to the vanishing growth of the non-epithelial tissue layers (Figure 7F). Prolonged growth of these layers simply leads to a prolonged time to reach the equilibrium crypt density. Thus, a crypt density-controlled contact inhibition of growth in the *SCs* can explain the growth of the intestinal epithelium in postnatal mice.

## 3. Discussion

Through the simulation of tissue dynamics, computational tissue models allow the causal linking of successive experimental observations. They have the advantage that they can be developed with a long-term view; if computational capacities are improved, further questions can be addressed based on the same model or straightforward model extensions. Exploiting these capabilities, computational tissue models have strongly supported the understanding of fate control of intestinal SCs in the last decade [42]. Here, we introduce an individual cell-based model of postnatal intestinal growth that builds on our previous models of the adult intestinal crypt [36,43] and intestinal organoids [16,44]. General results on intestinal postnatal growth obtained in simulation series were then utilized to link different experimental data on transgenic mice that express *Cd97* in the intestinal epithelium and develop a mega-intestine with normal microscopic morphology [8].

We provided evidence that in transgenic *Cd97* mice, the formation of the intestinal SC niche immediately after birth (P1–P7) and the following maturation of the intestine (P7–P14) are accelerated. This is somewhat counter to the formation of a mega-intestine seen in the adult transgenic mice. Around P14, when *Prdm1* is downregulated, several growth factors, known to be associated with crypt-fission and tissue growth during regeneration, become upregulated in the transgenic *Cd97* mice. These findings suggest an adaptive growth of the epithelium following accelerated intestinal growth. The regulation is an example of concurrency between autonomous and conditional fate specification induced by gene expression differences. Changes of tissue specification can be monitored, for example, by Klf4, which shows inverse regulation in Tg2 compared with the WT intestinal epithelium.

In our computational model, the expansion of the *SC* population is largely independent of the tissue growth rate and therefore not spontaneously adaptive. *SC* expansion is determined by the sensitivity to contact inhibition of growth, a cell-intrinsic property, and by *SC* niche biomechanics. Adaptive growth of the epithelium thus requires explicit synchronization between tissue growth and *SC* expansion (leading to crypt fission), not considered in the model. Similar morphology in transgenic *Cd97* and WT mice argues for a crypt density-controlled SC expansion. Such regulated adaptation might be realized by secretory activity of the epithelium and the cells of the submucosa as described by [11].

As crypt fission is initialized by separating clusters of PCs, we expect PC specification to be involved. A potential mechanism is increased PC specification at low crypt density. According to former model studies, high external Wnt or R-spondin enforces PC specification [16]. An isolated crypt surrounded by niche mesenchyme (e.g., sub-epithelial fibroblasts) that produces these factors [45] can attract more factors than competing crypts. Alternatively (or in parallel), a high concentration of noggin or gremlin1/gremlin2, also produced by the niche mesenchyme, represses bone morphogenic protein (BMP) [46] and therefore enforces niche expansion. This effect is also stronger at low crypt density.

The suppression of BMP in the villus mesenchyme can even result in *de novo* crypt formation in adult tissue [47]. Such behaviour has been modelled, applying Wnt and BMP diffusion–reaction systems [26]. However, *de novo* crypt formation contributes to the early postnatal intestinal phase only, whereas crypt fission is the dominant process later. Theoretical approaches predict a fixed wavelength of related tissue organization, depending on the biomechanical properties of the intestinal tissue [48]. Such organization is also seen in the first steps of our model simulations, and subsequent crypt growth based on those established structures.

In summary, we suggest the following scenario for the formation of a mega-intestine with an epithelium that shows WT morphology: First, tissue growth becomes accelerated by growth factors. In parallel, cell turnover of the crypts is increased to enable stable growth of the villi. Stable crypt density for different growth velocities suggests that this increase is ensured by the formation of new crypts via enforced fission. Enforced fission requires enforced SC expansion. We suggest that such enforced expansion originates from decreased sensitivity to contact inhibition of growth that is regulated by crypt density. In other words, we suggest a spontaneous adaptive behaviour of the epithelium following accelerated tissue growth.

Tissue growth has been linked to *Areg* and *Btc* expression [10]. Thereby, epithelial secretion might induce an entire cascade of signals that allows signal progression towards the outer layers such as the muscularis. According to our general tissue growth scenario, accelerated growth might originate either in a higher value of the initial growth rate r_G_ or in a larger value of the decay constant of growth τ_G_, due to increased growth factor expression. As long as the general growth scenario is conserved, tissue growth will eventually stop. It can partially reactivate after ileocecal resection but in the radial direction only [49]. Thereby, crypt density on the circumference remains conserved as well.

What ultimately stops SC expansion and thus crypt fission? Here, we suggest a crypt density-dependent mechanism regulating contact inhibition of growth. However, regulated pressure resistance of the BM or contractility of the fibroblast layer represent alternative scenarios.

During homeostasis, the epithelium changes from adaptive fission to adaptive proliferation. The pressure within the differentiated epithelium, which is reduced during the tissue growth phase, is increased and apoptosis on the villi starts. Increasing rates of apoptosis can be balanced by activating (accelerating) the proliferation of quiescent (slowly proliferating) intestinal SCs. The actual distribution of proliferating cells is determined by the rate of apoptosis. Massive apoptosis that exceeds the regenerative capacity can even result in villus contraction [7]. Cell loss is not balanced by crypt fission. It can be reactivated following crypt loss only, for example, as a consequence of radiation [50].

While the morphological differences between *Cd97* transgenic and WT mice vanish in adult mice, gene expression differences persist. Regardless, tissue growth stops. Does decreased sensitivity to contact inhibition of growth persist during homeostasis in adult transgenic *Cd97* mice? Results on EGFR-signalling might support this idea [51]. Decreased sensitivity to contact inhibition of growth does not change the distribution of proliferative cells but enables faster regeneration, because more cells per time unit can be provided. This would explain why Tg2 mice are more protected against DSS-induced colitis [52]. However, increased tissue pressure would change the morphology of crypts and villi, most probably increasing their size. This is not seen in transgenic *Cd97* mice, indicating that higher cell contractility is responsible for protection against colitis. Morphological changes are also present in other mice that develop a mega-intestine, for example, in *Cldn15* knock-out mice [9], indicating remaining changes in the growth balance in these mice.

Besides suggesting a postnatal growth scenario, our simulation results provide a hypothetical link between several phenomena induced or regulated by *CD97*, such as postnatal intestinal growth, DSS-induced colitis and tumour invasion. Further potential applications of the model are studies on how aberrant SC specification and BM biomechanics following mutations affect crypt formation and potentially lead to tumour precursor states as in familial adenomatous polyposis [53]. Simulation of the proposed crypt density-dependent control of crypt fission will require massively improved computational capacities. Clearly, as in any other computational model, the introduced model does not provide a one-to-one description of the tissue under consideration. Thus, the hypotheses generated about fate control during postnatal growth need to be validated experimentally. Here, model simulations can be utilized to suggest efficient experimental strategies.

## 4. Materials and Methods

### 4.1. Model of Neonatal Crypt Fission

The model builds on our former approaches to intestinal crypt and organoid dynamics. It describes populations of different cell types interacting with a basal membrane (BM). Cells are represented by elastic spheres and the BM by a triangulated network of semi-flexible polymers. In the following, we provide a brief description of the model. Further details and parameters are given in Supplement 2, Table S1.

As in our models of organoid growth [16,44], the BM is free to move and re-organizes permanently in response to cell-BM interaction. BM movement is the origin of tissue shape changes. BM re-organization, comprising the introduction of new triangles and the removal of existing ones, ensures that the mesh size of the BM is kept within a defined range. On one hand, this avoids penetration by cells from the epithelial or the sub-epithelial cell populations; on the other hand, it keeps the polymer number technically tractable.

The cell specification and differentiation of the model epithelium into subpopulations (*SCs* into *PCs*, *GCs*, *ECs*) follows the rules introduced in the crypt models [36,43]. Maintenance of *SCs* requires active Wnt and Notch signalling. Wnt signalling is activated either by external sources or by neighbouring *PCs* (number ≥ N_W_). Active Notch signalling requires contact to secretory cells (number ≥ N_N_). If the Wnt or Notch activity falls below certain thresholds, *SCs* specify. Such specification is reversible as long as progenitors do not become terminally differentiated. Low Notch induces *PC* specification, low Wnt *EC* specification, and low Notch together with low Wnt induces *GC* specification. To avoid the infinite expansion of an (*SC-PC*) niche population, *PC* specification requires sufficient positive tissue curvature C_0_; otherwise, pseudo-*GC* (with high Wnt) are specified.

Proliferation is modelled by a stochastic increase of the target cell volume V_T_ from V_0_ to 2V_0_ in N_P_ steps and a subsequent split of the cell into two daughters. The capability of proliferation is lineage-specific. *SC* are capable of permanent proliferation. *ECs* stop proliferation and become terminally differentiated at a fixed time t_prol_ after specification. *PCs* and *GCs* finish a last cell cycle after their specification before they become terminally differentiated. In extension to former crypt models, we introduce a population of sub-epithelial fibroblasts. These cells are capable of proliferation and adhere to the opposite side of the BM. They do not form subpopulations. All cells capable of proliferation are subject to contact inhibition of growth: they stop proliferating if they are compressed, and their actual volume V_A_ is smaller than (V_0_-dV). This regulation is fully reversible, that is, proliferation restarts if compression is released, as long as it has not been stopped by differentiation.

The initial tissue structure is cylindrical. This open structure mimics the crypt villus interspace that forms before birth. Details of *SC* specification associated with the initial model configuration are described in the text. All cells are free to move, driven by cell–cell and cell–BM forces. Cell–cell interactions are short-range neighbour interactions and include surface adhesion, mutual deformation and compression as well as attraction via an intercellular ‘apical’ polymer network. Cell–BM interactions are also short range and include adhesion via a selected BM-knot and repulsion via all others in the selected interaction range. Epithelial cells that cross the openings of the cylinder (villi) are removed from the system. In contrast, fibroblasts stop moving and remain at the openings. Cell apoptosis occurs when cells lose their contact to the BM. In contrast to our previous models, cells that lose contact to the BM do not undergo apoptosis immediately. These cells shrink rapidly in size, and during this process, the neighbouring cells experience a strong contraction via the intercellular network. This mechanism supports a closed epithelium. *PCs* have a limited average lifetime t_PC_.

### 4.2. Model Computations

Our simulations were performed in cooperation with the Centre for Information Services and High Performance Computing (ZIH) at the TU Dresden. These shared resources can be requested for each simulation with a maximum runtime of one week. Hence, depending on the number of crypts that form, we were able to simulate between 1 and 4 weeks of tissue development using an Intel Haswell CPU architecture with Intel(R) Xeon(R) CPU E5-2680 v3 processors and up to 256 GB RAM. For each setup we ran at least 10 simulations.

### 4.3. Data Analysis

We started analysis right after external Wnt sources were switched off (t = 0). This time point depends on the individual simulation. The various time frames denoted in our data plots refer to this variable time point and the corresponding ZIH run-time limits. For t > 0, observations were made every 1.2 h (observation step). This was sufficient to follow up crypt formation, cell turnover and lineage changes. For characterization of the turnover and *SC* numbers, we averaged the results of 20 observations. For turnover, we counted the epithelial cells that were removed at the cylindrical end only. Cells that lost contact to the BM were not considered.

The radial extension (along the cylinder axis) of the BM was determined with a resolution of 5 µm. Thereby, the BM was split into slices of 5 µm width, ignoring the open boundaries. For each point describing the BM, its distance to the cylinder axis was calculated as the radius. The average of those radii was finally considered as radial extension.

Calculation of the *SC* growth rate q was started at the time point where a linear fit over all following time points reached R > 0.95. For detecting the *SC* doubling time, t_d_ = ln(2)/(ln(10)q), only simulations were used in which non-interacting crypts form.

For *SC* compression analysis, we analysed the last 40 observation steps (2 days) of the simulations. Thus, for an average of 25 *SCs* per observation step, a set of about 1000 data points were analysed.

### 4.4. Mice and Ethics Statement

This research complies with the ethics guidelines of Leipzig University. We obtained ethics approval from the Landesdirektion Leipzig for i.p. injection of BrdU (TVV 52/13–06/Apr/2014, TVV 53/14–06/03/2015, TVV 22/16–01/06/2016). *Tg(villin-Cd97)2* mice (in short Tg2) expressed high-copy numbers of full-length mouse *Cd97(EGF1234)* under the control of the villin promoter [52]. Within the first week of life, we always analysed the same number of Tg2 and wild-type (in short WT) C57BL/6J offspring of one litter to ensure that postnatal Tg2 and WT mice had exactly the same age. Intestinal tumours were obtained from 12 month V*C^+^*^/*?*^*Msh2^LoxP^*^/*LoxP*^ (in short *Msh2^−^*^/*−*^) mice [19].

### 4.5. Intestinal Morphology

In neonatal mice up to P7, the whole intestine, and in older mice, intestinal segments [52], were dissected and paraffin-embedded. The crypt size was determined from microscope images taken from haematoxylin-eosin (HE)-stained sections using Fiji (www.fiji.sc, accessed on 20 June 2021). In neonatal mice, PC precursors were detected by their distinct eosinophilic staining, and later their typical granular pattern was used for identification. In 2- and 4-day-old mice, the number and position of proliferating BrdU-positive intestinal epithelial cells was determined as described [8]. Per 50 mg/kg body weight, 2 mg/mL BrdU (Sigma-Aldrich, Taufkirchen, Germany) was injected; i.p. Mice were sacrificed 2 and 24 h afterwards. Paraffin sections were stained for BrdU incorporation using the BrdU In-situ detection Kit II (BD Biosciences, Heidelberg, Germany).

### 4.6. Organoid 3D Live Imaging

Six centimetres of the jejunum was used to generate intestinal organoids [19]. Organoids were split 1 day before z-stack series were taken by 3D live imaging for 6 days. Organoid growth rates and the relative area A/A_0_ of the cross-section of growing organoids were quantified [19]. The growth pattern of each organoid was assessed at the various time-points either as branched, that is, multiple crypt-like structures project outward, or cyst-like, that is, hollow spheres with no or few projections.

### 4.7. Microarray and SOM Analysis

mRNA from jejunal samples of 2-, 3-, and 9-week-old Tg2 and WT mice (2 mice with 2 replicates each) as well as adult WT mice, organoids of adult WT mice (2 replicates), and tumours of 12 month old *Msh2^−^*^/*−*^ mice (4 replicates) were extracted using TRIzol (Thermo Fisher Scientific, Waltham, MA, USA) and analysed with the Bead Chip Array MouseRef-8 v2 (Illumina, San Diego, CA, USA). Data were selected by a *p*-value < 0.05 from the background corrected raw data. The base-10 logarithmic expression data were analysed by the oposSOM R package [54]. Data were filtered in order to consider only those IDs with reliable expression values (*p*-values < 0.05) for all samples. For the remaining 6365 IDs, log10 values were corrected for batch effects [55] and processed by the oposSOM-pipeline.

### 4.8. qRT-PCR

Intestinal tissue was homogenized in 600 µL RLT Buffer (Qiagen, Hilden, Germany) and total RNA was isolated according to the manufacturer’s instructions. For cDNA synthesis, 1 μg RNA was transcribed using the SuperScript™ IV First-Strand Synthesis System (ThermoFischer). Relative levels of mRNA were quantified using an Applied Biosystems 7500 Real-Time PCR System (ThermoFischer) with the ΔΔCt method. The expression of specific genes was normalized to mouse *Rps29*. Primer sequences are provided upon request.

### 4.9. Statistics Tests

*p*-values less than 5% were considered to be significant and are indicated with * *p* < 0.05, ** *p* < 0.01, or *** *p* < 0.001.

## Data Availability

The average expression data of three individual sample of each group (Figure 4) is available as supplementary material. This data includes gene symbols, the average expression as logarithmic fold change (logFC), rank scores (WAD, T-score, *p*-value) and the metagene position according to the gene’s location in the SOM.

## Supplementary Materials

The following are available online at https://www.mdpi.com/article/10.3390/ijms22147345/s1. References [56,57] are cited in the supplementary materials.
