# Supplementary material for: How to Obtain a Mega-Intestine with Normal Morphology: In Silico Modelling of Postnatal Intestinal Growth in a Cd97-Transgenic Mouse
Source: Int J Mol Sci. 2021 Jul 8;22(14):7345. doi: 10.3390/ijms22147345 (PMC8305140; doi:10.3390/ijms22147345)
Supplement: Supplementary file 1 [file ijms-22-07345-s001.zip › ijms-1264762 suppl. revised.pdf]

Supplement 1: Details on experimental results

Intestinal morphology and organoids from Tg2 mice. The intestines of Tg2 mice grow faster and their cells show premature differentiation (Fig. A1). A detailed quantification of their morphological and cellular properties can be found in [8].

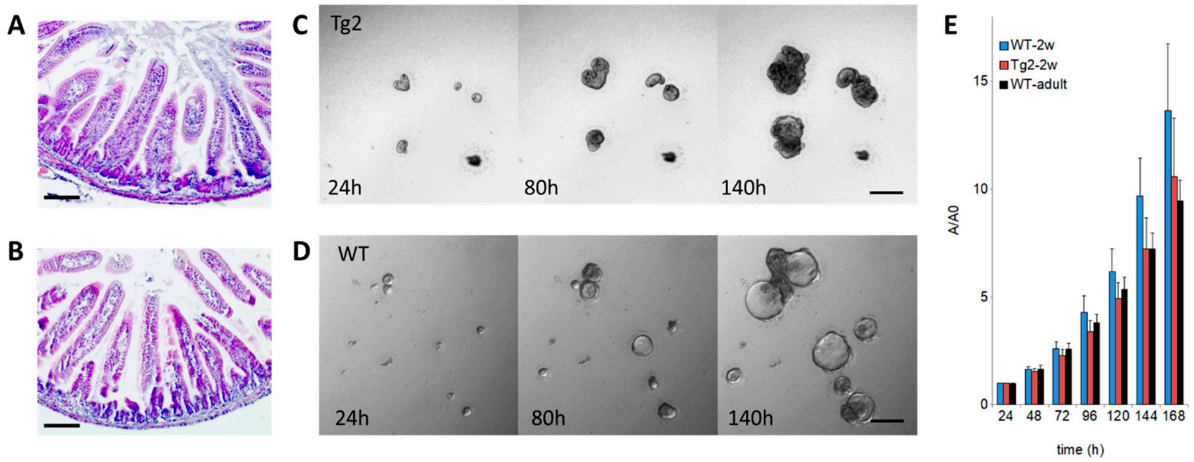

Figure S1: Characteristics of the intestine and organoids from 2-week-old Tg2 mice. A, B) The postnatal intestines of Tg2 mice (A) show similar microscopic morphology with WT mice (B) (statistics see: [8]). Representative images of HE-stained jejunal sections of 2-week-old Tg2 and WT mice (scale bars 100 µm). **C, D)** Organoids from two-week-old Tg2 mice (C) more frequently show a branched growth pattern compared to WT mice (D). Representative time series of organoid growth (scale bars 200 µm). **E)** Comparison of the growth rates of organoids from 2-week-old Tg2 and WT mice with those of organoids from adult WT mice as provided by [19].

Self-organizing map (SOM) analysis of microarray data obtained from intestinal samples. SOM analysis identified 22 meta-gene spots based on correlated gene expression (Fig. A2). The similarity of gene regulation in Tg2-2w with adult mice manifests in genes of spots A and B. This early expression of genes in Tg2 mice, which usually peak later in development, is remarkable. The regulation is much stronger than that of spot C genes that are upregulated in the intestine of WT-2w mice and organoids, i.e., of genes confirming similarities between the juvenile intestine and intestinal organoids [18].

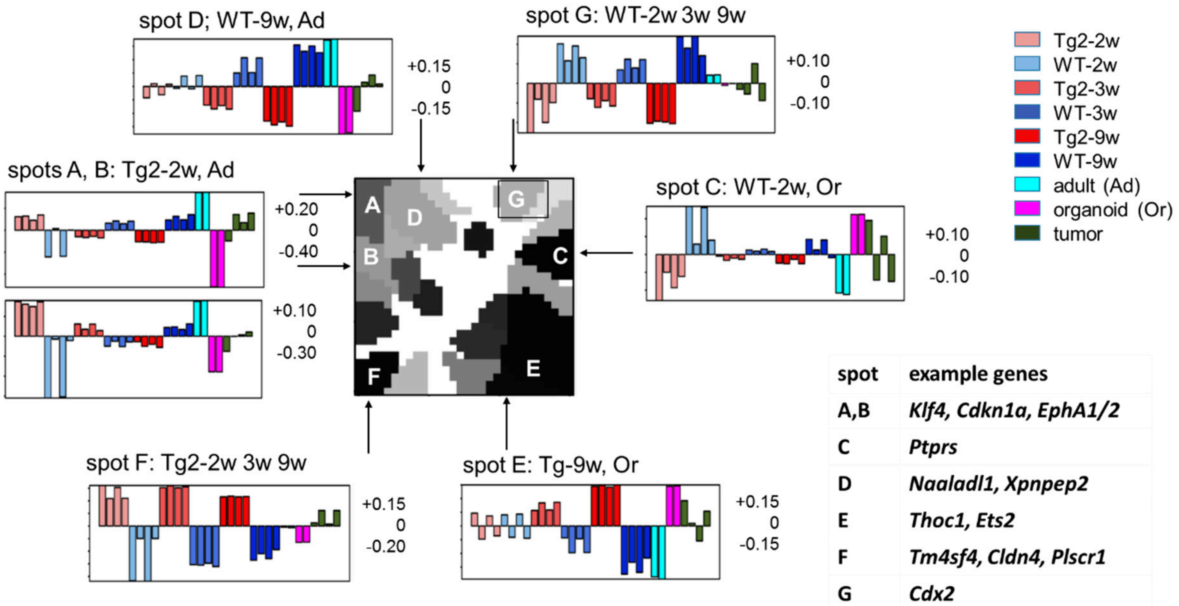

spot	example genes
A,B	*Klf4, Cdkn1a, EphA1/2*
C	*Ptprs*
D	*Naaladl1, Xpnpep2*
E	*Thoc1, Ets2*
F	*Tm4sf4, Cldn4, Plscr1*
G	*Cdx2*

Figure S2 (previous page): SOM meta-gene spots based on microarray data. Genes activated in Tg2-2w and adult WT (WT-A) mice only are found in spots A and B. Those activated in WT-2w and organoids (O) only accumulate in spot C. Genes specifically activated in WT-9w and adult WT mice refer to spot D. Those genes specifically activated in Tg2-9w mice and organoids (O) contribute to spot E. Genes activated in Tg2 and WT mice only contribute to spot F and G, respectively. The GSZ-score of all groups (see legend) is shown in the boxes. Examples of genes associated with the spots are provided in the table.

Adult Tg2 mice adopt an immature EC expression profile. Spot D genes are activated in 9-week-old (WT-9w) and adult WT mice. Thus, they represent a part of the differentiation program of the WT small intestine (Fig. A3). Consistently, spot D comprises genes expressed by mature ECs of the small intestine (e.g., *Naaladl1*, *Xpnpep2*). Spot D genes enriched in the GO sets “epithelial differentiation” and “brush border”. In 9-week-old Tg2 (Tg2-9w) mice and organoids, these genes are repressed, indicating incomplete differentiation. Spot E genes show an inverse expression compared to spot D. They are enriched in genes (e.g., *Thoc1*, *Ets2*) preferentially expressed by intestinal progenitors and transient amplifying cells (Fig. A3). Functional annotation of spot E genes yields a strong enrichment in the GO set “ribonucleoprotein complex biogenesis” [56]. These results indicate that regardless of its premature state in the first weeks after birth, Tg2 tissue adopts regulatory states characteristic for a rather incompletely differentiated intestine, similar to intestinal organoids, in the following weeks.
Spot F genes that are upregulated in Tg2-2w, 3w, 9w but downregulated in age-matched WT mice are described in the text (and below). Spot G comprises genes that are downregulated in Tg2-2w, 3w, 9w but upregulated in age-matched WT mice. Spot G shows no cell type-specific expression. Most of the genes repressed in Tg2 show weaker expression differences between Tg2 and WT tissue, indicating that transgenic *Cd97* results preferentially in gene activation.

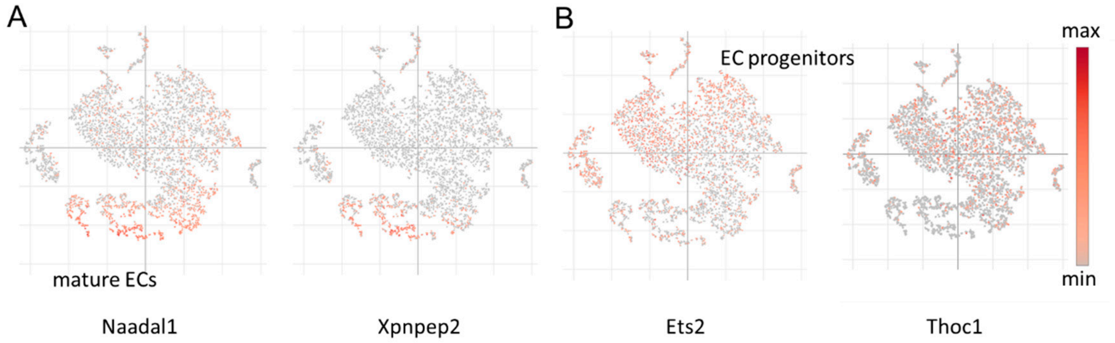

Figure S3: Immature EC expression profile in Tg2 mice. Re-analysis of published single cell RNA-sequencing data [30]. Each point represents one analyzed intestinal cell, which clusters according to its transcriptomic profile. The expression level of the indicated gene is shown for each cell.
A) Spot D genes, repressed in Tg2 mice, are preferentially expressed by mature ECs. **B)** Spot E genes, activated in Tg2 mice, are preferentially expressed by EC progenitors with respect to transient amplifying cells.

Enhanced expression of secretory cell markers in Tg2 mice. Spot F genes comprise markers of secretory cells such as *Tm4sf4* and *Clnd4.* In contrast to *Areg* and *Btc*, they are activated in adult WT tissue but not in organoids derived from WT mice (Fig. A4), suggesting that they represent a part of the premature signature of Tg2 tissue. However, this was not confirmed by qRT-PCR (Fig. A4), demonstrating that these genes are not expressed before *Prdm1* downregulation.

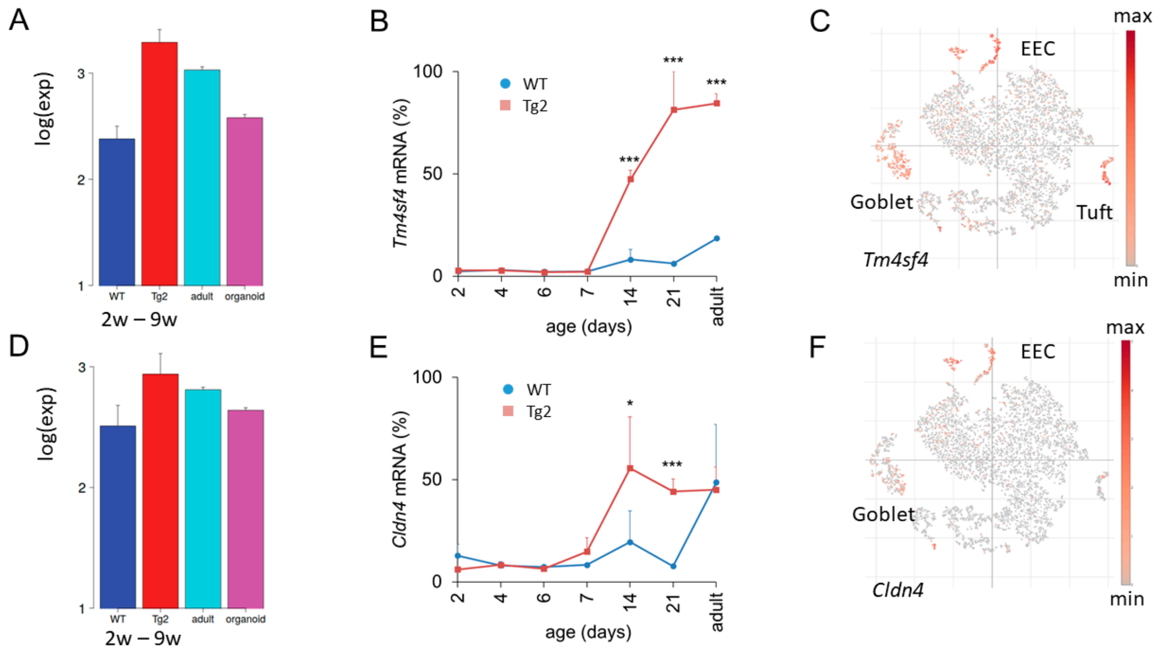

Figure S4: Enhanced expression of secretory cell markers in young Tg2 mice. **A-C)** Expression of *Tm4sf4* in Tg2 and WT mice. **A**) *Tm4sf4* microarray data of intestinal jejunal samples of young WT and Tg2 mice, adult WT mice and WT organoids. **B)** *Tm4sf4* qRT-PCR levels (n=3 mice/genotype/age, normalized to *Rps29*, mean ± SD, ***p<0.001). Tg2 compared with WT. **C)** In the adult WT intestine, *Tm4sf4* expression is restricted to enteroendocrine cells (EEC) and goblet cells (GC); re-analysis of published single cell (sc)RNA-sequencing data [30]. Each point represents one analyzed intestinal cell, which clusters according to its transcriptomic profile. **D-F)** Data as in A-C) for *Cldn4.*

Supplement 2: Details on simulation results

Our model allows for the simulation of growth rate r between 0 and 0.5 µm/day. While for 0.1 µm/day, BM network deformation due to cell-BM interaction dominates tissue shape changes, radial expansion is dominant for 0.5 µm/day (Fig. A5). Simulations show a considerable variance of growth behavior. This variance originates in the different number of crypts formed (Fig. A6), affecting the 'time to equilibrium'. Stationary expansion of the *SC* pool is observed until the crypts start fission.

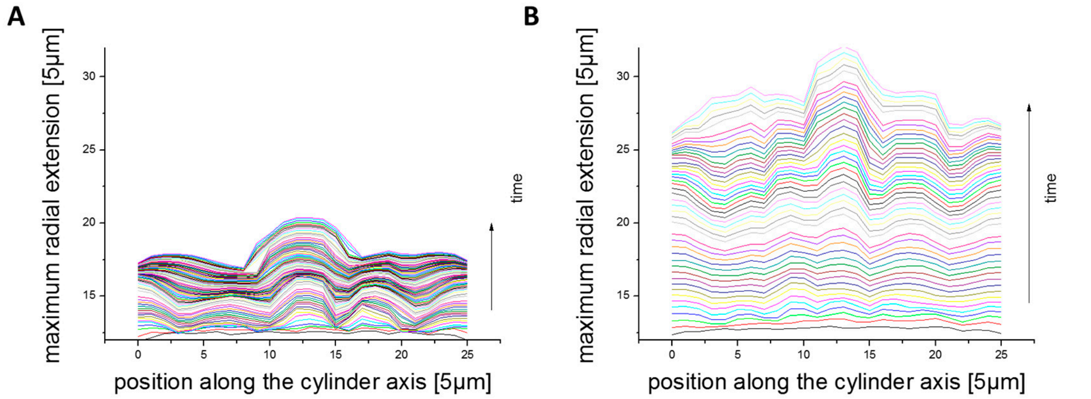

Figure S5: The tissue growth process. Shown are profiles of the maximum radial extension of the polymer network for different time points for r=0.1 **(A)** and r=0.5 **(B)**. The time between two profiles is 6h. Shown is the development for 12 days. For r=0.1, changes are dominated by crypt growth, while for r=0.5, external growth prevails.

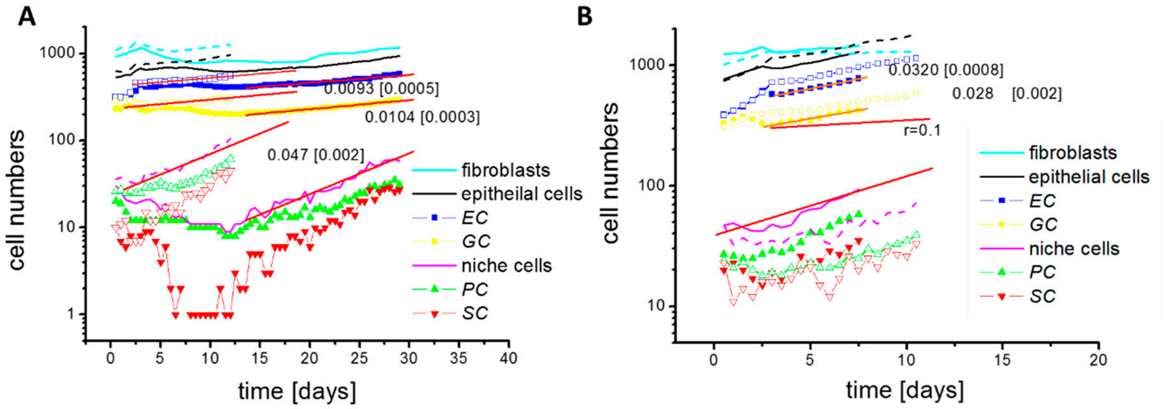

Figure S6: Cell numbers. Shown is the size of different cell populations for growth rates r=0.1 (A) and r=0.5 (B). **A)** Inter-Simulation-Variance (slow growth, r=0.1): Compared are two simulations with identical parameters (see also Fig. 7A), where one (solid lines, filled symbols) and five crypts (dashed lines, open symbols) are formed. The slopes [sd] within the expansion phase are fitted for the 'one crypt' simulation and visually compared with the 'five crypt' simulation. **B)** Turnover effects (fast growth, r=0.5): Compared are two simulations with identical parameters but with ongoing (solid lines, filled symbols) and blocked turnover (dashed lines, open symbols) onto the villi. While the slope for niche cell growth (*PCs*+*SCs*) is comparable with simulations shown in (A), *ECs* and *GCs* grow significantly faster. However, the slope is only 3 times larger, indicating a reduced cell density in the system compared to (A) due to faster expansion. All simulations were run with the same maximum CPU-time. The growth rate r=0.5 is at the limit of our simulation power, allowing an analysis of about one week growth time.

Mathematical details of the model.
Cells are modeled as individual objects that can adhere to other cells or the BM and can be deformed and compressed. In general, their properties depend on the cell type. In the following, the equations for the cell-cell and cell-BM interaction energies and for the system's force balance are provided. Afterwards, the BM-model is described.

Cell-cell interaction energies. Cell interactions are described by center-center pair interaction potentials and depend exclusively on the distance $y_{i,j}$ between cell i and j. Except for the cell 'apical network' potential, the potentials are zero for distances $y_{i,j} > R_i + R_j$, where R_i and R_j are the radii of cell I and j, respectively.

Cell adhesion: $W_i^A = \varepsilon_c \sum_j A_{i,j}^C$

Here, ε_c is the cell-cell adhesion constant and $A_{i,j}^C$ the contact area between cell i and j. The sum runs over all neighbors.

Cell deformation: $W_i^D = \sum_j \frac{2y_{i,j}^{5/2}}{5D_{i,j}} \sqrt{\frac{R_i R_j}{R_i + R_j}}$

with $D_{i,j} = \frac{3}{4}\left(\frac{1-\nu_i^2}{E_i} + \frac{1-\nu_j^2}{E_j}\right)$. Here, ν_i is the Poisson ratio and E_i the Young modulus of cell i.

Cell compression: $W_i^C = \frac{K}{2V_i^T}\left(V_i^T - V_i^A\right)^2$

Here, V_i^T and V_i^A are the target and the actual volume of cell i, respectively. K is the bulk modulus of the cells. Epithelial cells are inter-connected via an 'apical' network of semi-flexible polymers of n^A segments with an equilibrium lengthy_0^A. The number n^A is chosen such that: $y_0^A < y_{0,max}^A$.

Elastic energy: $W_k^N = \frac{n^A E^A}{2}\left(y_k - y_0^A\right)^2$

Bending energy $W_{k,k+1}^B = K^A\left(1 - cos\left(\gamma_{k,k+1}\right)\right)$

Here, y_k is the length of segment k of the polymer (connecting cells i and j). The equilibrium polymer length $n^A y_0^A$ is the distance between the cells at the time point when they become inter-connected. $E^A$is the spring constant, K^A is the bending modulus and $\gamma_{k,k+1}$ is the angle between two adjacent segments. Cell-cell connections are cut if one y_k exceeds a threshold distance y_{AP}, i.e., the net cannot resist higher forces and ruptures. Connections are also cut if a growing cell reaches the volume V_{AP}. This enables reorientation and formation of new connections during cell division. If a cell undergoes apoptosis, y_0 is reduced, K_P is increased, and the target cell volume V_T decreases, supporting a closed epithelium.

Cell-BM interaction energies. Cells interact with the BM via the knots of the net that are closer than the cell radius R. They adhere to the knot with the maximum adhesion energy and are repelled by all other knots. This assumption ensures that cells can move without adhering to dense regions of the net.

Cell-knot adhesion: $W_{i,k}^{BM} = \varepsilon_{adh}\left(\varpi_{adh} ln\left(\frac{y_{i,k}}{R_i}\right) - \frac{y_{i,k}}{R_i}\right)$
Cell-knot repulsion: $W_{i,k}^{BM} = \varepsilon_{rep}\left(ln\left(\frac{y_{i,k}}{R_i}\right) - \frac{y_{i,k}}{R_i}\right)$

Here, $\varepsilon_{adh}$and ε_{rep} are interaction constants, and ω_{adh} ($0< \omega_{adh} <1$) is a parameter defining the position of the maximum adhesion energy.

Force balance. The deterministic forces F_i^D and G_i^D that change cell position and radius, respectively, are calculated from the interaction energies. A friction dominated regime is assumed; for each cell i, friction forces F_i^f (G_i^f) balance the deterministic forces $F_i^D(G_i^D)$ and stochastic forces F_i^S (G_i^S).

Cell position: $F_i^f = \eta_{cc} \sum_j A_{i,j}^C (v_i - v_j) +$

Here, $\eta_{c,c}$ is the cell-cell friction constant, $\eta_{c,BM}$ the cell-BM friction constant, η_E the cell-environment friction constant, and v_i and v_j are the velocities for changing the position of cell i and cell j, respectively. The sum runs over all neighbor cells. For simplicity, the same the friction constant $\eta_{c,c}$ for movement parallel and normal to the contact area is assumed. The velocity of the knots of the BM is considered to be small compared to the cell velocities and, thus, is neglected.

Cell radius: $G_i^f = \eta_{cc} \sum_j A_{i,j}^C (w_i + w_j) + \left(\sum_k \eta_{c,BM} + \eta_V\right) w_i = G_i^D + G_i^S$

Here, η_V is the friction constant for volume changes, and w_i and w_j represent the velocities of changing the radius of cell i and j, respectively. While translation requires reorganization of the adhesive knot, volume changes affect all knots in contact. Thus, the second sum runs over all knots in contact with cell i.

BM biomechanics. The BM is constructed in two steps. First, a triangulated mesh is generated by MeshLab (Visual Computing Lab). Afterwards, all edges are replaced by semi-flexible polymers. The maximum equilibrium segment length $l_{0,max}^B$ is varied until a narrow distribution of the segment length with an average equilibrium length$l_0^B \;\; _{av}$ is obtained. The stretching and bending energies of the polymers are given by:

$$w_k^{elast} = \frac{E^P}{2}(l_k - l_0^B)^2$$

$$w_{k,k+1}^{bend} = K^P \left(1 - cos(\gamma_{k,k+1})\right)$$

Here, $E^P$is the spring constant and K^P is the bending modulus of the polymers. The parameters of the polymers depend on the cell type in contact with it. In the simulations presented, PCs decrease the spring constant. The bending of adjacent surface elements of the net is associated with the energy:

$$w_{m,m'}^{curv} = K^S \left(1 - cos(\alpha_{m,s})\right)$$

Here, K^S is the bending modulus of the surface and $\alpha_{m,m'}$ is the angle between surface normal of the adjacent elements m and m'.

Cell-BM and intra-network interaction forces the knots of the network to move. Thereby, a friction force F_k^{BM} balances the deterministic forces on each knot k of the network, including the friction force related to the movement of all cells in contact with k.

$$F_k^{BM} = \eta_{BM} v_k$$

Here, η_{BM} is the friction constant controlling movement of knot k with velocity v_k. In addition to these changes, the basal membrane expands radially along the cylinder axis by external forces. The radial distance of knot k from the cylinder axis increases by a defined rate Γ.

BM reorganization. Deformation of the net by cell-BM forces change the mesh sizes A_M. In case A_M exceeds a threshold A_M^u, new triangles are constructed. In case A_M falls below a threshold A_M^l, it is fused with neighboring triangles.

Table S1: Parameter Set

Symbol	Value	Parameter	References
**Parameter of the cell model**			
V_0	4/3π $(5\mu m)^3$	minimal volume of an isolated cell	Estimated, $R(V_0)=5\mu m$
τ	10 - 14 h	intrinsic cell growth time	leads to realistic cycle times: 18-24h
E	1 kPa	Young modulus	[57]
K	1 kPa	Bulk modulus	
ν	1/3	Poisson ratio	
ε_c	200 μN/m	cell-cell anchorage	
V_p	0.85 -0.95 V_0	threshold volume regarding contact inhibition	specified in the text
**Parameter of the BM**			
l_0^B av	~0.75 μm	average intrinsic length of a polymer segment	Adjusted
$A_M{}^u$	1.25 pm^2	area at which a triangle is subdivided	set: prevents penetration of the net
$A_M{}^l$	1.00 pm^2	area at which a set of triangles is joined	set: prevents accumulation of flat triangles in folded areas
E^P	5 mN/m	elastic modulus of the polymer segments	[44]
E^P_{PC}	0.1 mN/m	elastic modulus of the polymer segments in contact with PCs	
K^P	100 fNm	bending modulus of the polymer segments	
ω	0.96	threshold number cell-BM potential	
K^S	80 fNm	bending modulus of the BM	four times the value used by [44], supports crypt stability
ε_{adh}	15 pNm	Cell-knot interaction energy (adhesive knot)	set: required to ensure a cell detachment <5% per day
ε_{rep}	0.02 pNm	Cell-knot interaction energy (repulsive knot)	set: required to avoid penetration of the net
R	0- 5 μm/day	Expansion rates in radial direction	Set: specified in the text

**Friction Parameters**			
$\eta_{C,C}$	50 GNs/m^3	friction constant for cell-cell friction	[57]
η_V	400 Ns/m	friction coefficient for volume compression	
η_E	0.4 Ns/m	friction coefficient of the medium	
$\eta_{C,BM}$	3.0 Ns/m	friction coefficient for cell-BM friction	[36]
$\eta_{PC,BM}$	8.6 Ns/m	friction coefficient for PC-BM friction	
η_{BM}	8.6 Ns/m	friction constant for BM motion	
**Parameter of the lineage specification and differentiation model**			
N_W	1	Number of Wnt secreting neighbors required for SC maintenance	[16]
$N_{N,SC}$	2	Number of Notch-ligand providing neighbors for SC maintenance	
$N_{N,EC}$	1	Number of Notch-ligand providing neighbors for EC maintenance	
C_0	4 10^4 /m	mean surface curvature required for PC-specification	curvature radius (25µm) close to the crypt radius (~20µm)
t_{PC}	3 weeks	average lifetime of PCs	[43]
t_{PC}^{Apop}	12h	Average lifetime of PCs without contact to SCs or ECs	
t_{prol}	48h	proliferation activity before terminal differentiation after specification	Set
**Parameters of the apical net**			
$l_{0,max}^A$	5 µm	maximum intrinsic length of a polymer segment	Adjusted
K^A	10 fNm	bending modulus of the apical net polymers	Set
E^A	0.2 mN/m	elastic modulus of the apical net polymers	Set
y_{AP}	2 x (6.1µm)	maximum distance for connections in the apical net	two times S-phase radius
V_{AP}	1.85 V_0	threshold cell volume for detachment from the apical net	S-phase volume
E_{apop}^A	10 mN/m	elastic modulus of the apical polymers of an apoptotic cell	Supports closed epithelium. Cells are removed at: $V_T=0.1V_0$.
y_{apop}	0.3 y_0	reduced intrinsic polymer length of an apoptotic cell	
v_{apop}	$(V_0/2)/h$	shrinking rate of V_T during apoptosis	

Additional References

56. Pitzonka L, Wang X, Ullas S, Wolff DW, Wang Y, Goodrich DW. The THO ribonucleoprotein complex is required for stem cell homeostasis in the adult mouse small intestine. Mol Cell Biol. 2013; 33(17):3505-14.

57. Galle J, Loeffler M, Drasdo D. (2005). Modeling the effect of deregulated proliferation and apoptosis on the growth of epithelial cll populations in vitro. Biophys J., 88:62-75
